# Ecological and health risks from heavy metal sources surrounding an abandoned mercury mine in the island paradise of Palawan, Philippines

**DOI:** 10.1016/j.heliyon.2023.e15713

**Published:** 2023-04-28

**Authors:** Reymar R. Diwa, Custer C. Deocaris, Lhevy D. Geraldo, Lawrence P. Belo

**Affiliations:** a Research and Development Center, Rizal Technological University, Mandaluyong City 1550, Philippines; b Atomic Research Division, Philippine Nuclear Research Institute, Department of Science & Technology, Diliman, Quezon City 1101, Philippines; c Earth and Space Sciences Department, College of Arts and Sciences, Rizal Technological University, Mandaluyong City 1550, Philippines; d Department of Chemical Engineering, De La Salle University, Manila 1004, Philippines; e BANToxics, Barangay Central, Quezon City, 1100 Philippines

**Keywords:** Abandoned mine, Heavy metals, Multivariate analysis, Public health and ecological risk assessments, Sediment and soil pollution

## Abstract

A recent survey that determined heavy metal concentrations in an abandoned Hg mine in Palawan, Philippines, found the occurrence of Hg with As, Ba, Cd, Co, Cr, Cu, Fe, Mn, Ni, Pb, Sb, Tl, V, and Zn. While the Hg originated from the mine waste calcines, the critical knowledge about the origin of the other heavy metals remains unknown. This study assessed the ecological and health risks from heavy metal pollution surrounding the abandoned Hg mine. Principal component analysis (PCA) showed that the abandoned mine and natural sources (i.e., local geology) are the two main contributors of heavy metal pollution. Historically, the mine waste calcines (retorted ore) were used as construction material for the wharf and as land filler for the adjacent communities. There is highly strong ecological risk associated with the heavy metals: Ni, Hg, Cr, and Mn contribute 44.3%, 29.5%, 10.7%, and 8.9% to the potential ecological risk index (RI), respectively. Hazard index (HI) exceeded 1 for both adults and children in all the sampling locations, implying non-carcinogenic adverse effects. The total cancer risk over a lifetime (LCR) also exceeded the threshold limit of 10^−4^ for both adults and children, contributed mainly by Cr (91.8%) and As (8.1%). By combining the results of the PCA and risk assessments, a clear link between heavy metal source apportionment to ecological and health risks was established. It was estimated that the abandoned mine contributed to most of the ecological and health risks for people living near the wharf that was built using the calcine, as well as the nearby Honda Bay. The findings of this study are expected to help policy makers develop regulations that will safeguard the ecosystem and the general public from the damaging impacts of heavy metals from the abandoned mine.

## Graphical abstract

**Figure:**
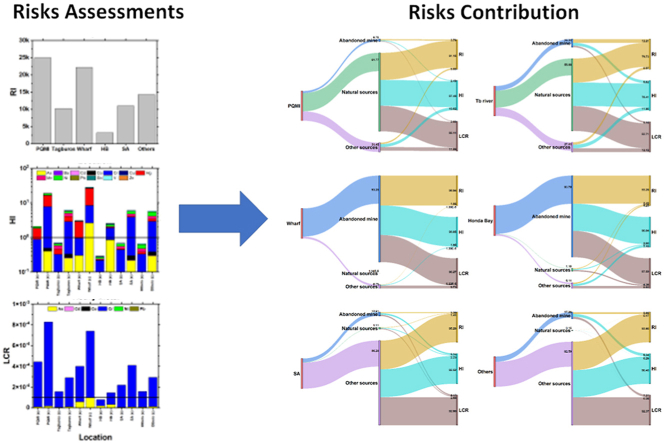

## 1 Introduction

Palawan Province is the largest province in the Philippines with a total area of 14,649.73 km^2^ and is composed of over 1700 islands: Calamian Island group in the north, Cuyo Island group in the northwest, and the Balabac-Bugsuk group in the southwest. Famously known as an island paradise in the global tourism sector, Palawan is also the Philippine's biodiversity corridor and the last ecological frontier being refuge to an immense biodiversity of corals, seagrass meadows, mangroves, marine mammals, freshwater fishes, amphibians, reptiles, birds, and terrestrial mammals, many of which are endemic if not threatened [1,2]. In 1990, UNESCO also designated Palawan as a “Man and Biosphere Reserve” [3].

There is also an abundance of mineral reserves in Palawan. In a geochemical survey, ores of Ni, Cr, Au, Cu, Co, Hg, and rare earth elements abound in Palawan [4] indicating that the island is an ideal site for conducting mining operations. In fact, in the past decades, regional economic development was contributed largely by mining operations of multinational companies, including the Rio Tuba Nickel Mining Corporation (RTNMC) [5]. As of this writing, open-pit mining activities for laterites in Palawan are still operating in the southern part of the main island. With the need to boost the local economy in response to COVID-19, it is expected that the national government will allow new mining concessions in the province [6].

The case of Palawan Island has been instrumental in strengthening environmental protection from unregulated mining practices in the country. Various legislative and policy instruments were passed including Republic Act 7942 (Philippine Mining Act of 1995), Department of Environment and Natural Resources (DENR) Administrative Order No. 2010–21 (Mining Act IRR), and Executive Order No. 79, s. 2012 (Institutionalizing and Implementing Reforms in the Philippine Mining Sector, Providing Policies and Guidelines to Ensure Environmental Protection and Responsible Mining in the Utilization of Mineral Resources) [7]. While the local government and mining companies have been working to address the long-term environmental effects like erosion, biodiversity loss, and contamination of groundwater by chemicals from the mining processes, there are still abandoned mines, like that of the Palawan Quicksilver Mines, Inc. (PQMI), that need to be rehabilitated. Historically, PQMI extracted a huge amount of mercury (Hg) deposits through open-pit mining from 1953 to 1976 in which around 2900 metric tons of mercury and 2,000,000 metric tons of mine waste calcines were produced. The mining operation stopped in the 1970s due to the declining price of mercury in the world market and the mining site was abandoned with no plans of rehabilitation [8]. Remnants from the mining company operations, such as the mine waste calcines, were used to build the peninsula or jetty in Honda Bay, the artificial lagoon, and some landfills. Calcine is a waste by-product after the smelting of the ore to extract the Hg. Through time, with weathering and erosion, mercury seemed to infiltrate the aquatic environment of Honda Bay [9,10]. Moreover, heavy metals attached to fine, dry particles which could be present in the abandoned mine can end up in the atmosphere, posing serious ecological and human health risks [11].

While existing studies were conducted to assess the extent of mercury contamination in the surrounding area of the abandoned mine [9,10], detailed health and environmental impacts of *admixture* of the heavy metals, their possible origins, and the relative contribution of the sources to the risks remain underassessed. In this study, the ecological and human health risks (non-carcinogenic and carcinogenic) associated with the heavy metals were calculated and combined with the results of the multivariate analysis, i.e., Principal Component Analysis (PCA), to estimate the risk contribution of pollution sources. To our knowledge, this is the most comprehensive study to date on the long-term ecological and health impacts and source apportionment of heavy metals surrounding the PQMI abandoned mine in Palawan, a setting top billed as “one of the most beautiful islands in the world”. Policy recommendations from the analysis are provided.

## 2 Materials and methods

### 2.1 Study area

**Fig. 1:**
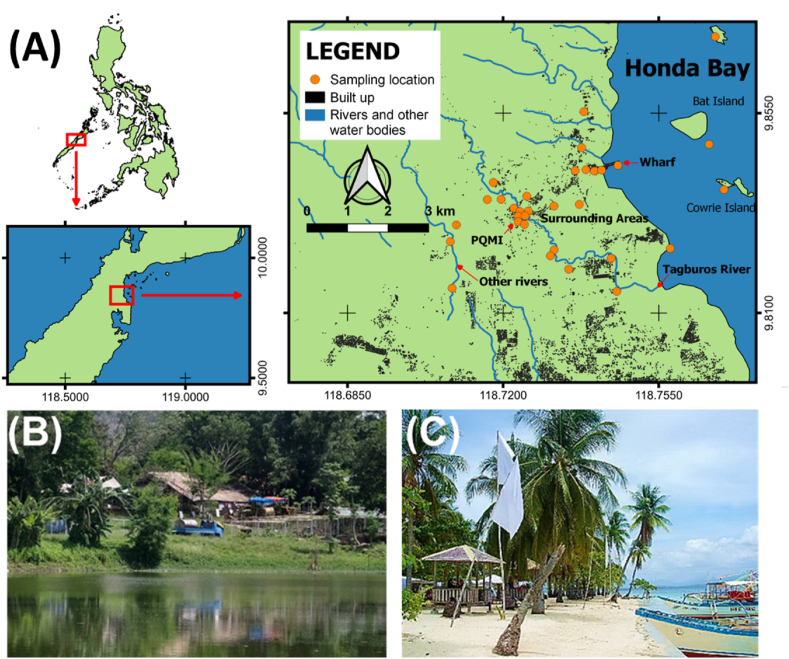
Sampling areas. (A) As modified from Samaniego et al. (2021), maps showing the site where samples were collected by Samaniego et al. (2021): PQMI pit lake (n = 4), Honda Bay Wharf (n = 3), along Tagburos River (n = 9), Surrounding Areas (n = 6), along the coast of Honda Bay (n = 6), and other rivers (n = 4). Also, photographs of (B) the PQMI lake and (C) Honda Bay are shown. Pictures of the lake and bay are respectively credited to government websites, Philippine News Agency (www.pna.gov.ph) and the local government of Puerto Princesa, Palawan (www.puertoprincesa.ph).

The study area, covering the villages of Santa Lourdes and Tagburos, is located 14 km north of Puerto Princesa City, the capital of Palawan. The villages are home to more than 12,200 people in 2015. The soil and sediment samples collected and analyzed by Samaniego et al. [10,12] are from the following areas: PQMI, Tagburos River, wharf, Honda Bay, surrounding areas, and other rivers are shown in Fig. 1A. The open-pit mining of cinnabar for Hg by PQMI resulted to the mercury-tainted artificial lake designated as PQMI pit lake (Fig. 1B). Mine waste calcines produced during the operation of the mining company were used to construct the wharf at Honda Bay as a former port for the company's mining operations. Now abandoned, the local government repurposed the wharf for tourist and fishing boats to access adjacent islands like Cowrie and Bat Islands. The village of Sitio Honda Bay was also built from the mine tailings [10,12]. PQMI pit lake is traversed to the south by the Tagburos River which drains to Honda Bay (Fig. 1C). The numerous islands adjacent to the bay lodge are world-famous resorts and sites for snorkeling and scuba diving. Honda Bay is also a rich fishing ground for small-scale fisherfolks and commercial fishing companies.

### 2.2 Sediment heavy metal data

The most recent dataset of elemental compositions of surface soils and sediments from Samaniego et al. [12] were analyzed. A total of 32 samples were collected by Samaniego et al. from September 2018 to October 2019 at the following sites: soil samples from PQMI, the surrounding areas, and Honda Bay wharf; and sediment samples from Tagburos river, other rivers, and Honda Bay coast (Fig. 1A). Details of the analysis have been reported by Samaniego et al. [12]. Briefly, approximately 1 g of the sample was digested in ionic solutions using a combination of HNO_3_, HF, HClO_4_, and HCl and then analyzed for heavy metals using Inductively Coupled Plasma Mass Spectrometry (ICP MS). To assure the quality of the analysis, a certified reference material (OREAS 44P) with the desired range of heavy metal concentrations were used for quality control. Meanwhile, total Hg was analyzed using U.S. EPA Method 7473 in direct mercury analyzer DMA-80 evo. The quality of the analysis was guaranteed by using certified reference material for stream sediments (GSD-1). Data on the mean heavy metal concentrations of As, Ba, Cd, Co, Cr, Fe, Hg, Mn, Ni, Pb, Sb, Tl, V, and Zn of the samples, excluding the mine waste calcines, were analyzed in this study.

### 2.3 Multivariate analysis

To identify the sources and apportionment of the heavy metals, PCA was performed using R ver. 4.0.4. PCA reduces the dimensionality of several intercorrelated quantitative variables into a new set of variables, called principal components, without significant loss of data [13,14]. The “PCA” syntax in R was used to calculate the eigenvalues, proportion of variance, and elemental contribution and apportionment of the principal components.

### 2.4 Ecological risk assessment

The ecological risks associated with the heavy metals were assessed using the potential ecological risk index (RI). RI measures the vulnerability of organisms to heavy metal contamination [15]. It is calculated using Eq. (1),

(1) $$RI=\sum_{i=1}^{n}{Er}^{i}=\sum_{i=1}^{n}{Tr}^{i}\times {Cf}^{i}$$

where *Er*^*i*^ is the potential ecological risk factor of the heavy metal, Tr^*i*^ is the biological toxic response factor of the heavy metal (*Tr*^*i*^: 40 – Hg, 30 – Cd; 10 – As; 5 – Cu, Ni, Pb; 2 – Cr, V; 1 – Zn), and *Cf*^*i*^ is the contamination factor of the individual heavy metal [16]. RI is rated as < 150 low risk; 150–300 moderate risk; 300–600 considerable risk; and >600 very high risk. The *ER*^*i*^ of the individual heavy metals are classified as < 40 low risk; 40–80 moderate risk; 80–160 considerable risk; 160–320 high risk; and >320 very high risk [15,[17], [18], [19]].

### 2.5 Human health risk assessments

Risk assessment provides a mechanism for structured data analysis to estimate health or environmental outcomes [20,21]. Except for Fe and Tl [[22], [23], [24], [25]], the heavy metals analyzed in this paper have toxicological and carcinogenic effects on humans. Assessment of non-carcinogenic and carcinogenic risks from heavy metal exposure of adults and children at the study sites made use of the recommended guidelines by United States Environmental Protection Agency (USEPA) [26,27]. These indices quantify the human health risks of heavy metal contamination in soil via ingestion, dermal, and inhalation exposures pathways.

Chronic Daily Intake (CDI) is the dose received through each of the heavy metal exposure pathways [25,28]. Hazard Quotient (HQ) is the measure of the potential non-carcinogenic toxicity to occur to an individual due to exposure to heavy metals. The risks associated with heavy metals are additive [29]. Thus, the Hazard Index (HI) was computed as the sum of the HQ of the individual heavy metals for the three exposure pathways. An HI > 1 indicates the probability of developing non-carcinogenic effects which tends to increase with the value [28,30]. Carcinogenic Risk (CR) estimates the probability of developing cancer for individuals because of exposure to carcinogenic metals. The total cancer risk over lifetime (*LCR*) is the sum of the CR by the individual heavy metals. The range of LCR can be characterized as follows: very low (<10^−6^); low (10^−6^–10^−5^); medium (10^−5^–10^−4^); high (10^−4^–10^−3^); and very high (>10^−3^) [31]. CDI, HI, and LCR are calculated using Equations (2), (3), (4), (5), (6), (7), (8)). The definition of variables and their values are summarized in Table 1.

**Table 1:** Values of variables for the assessment of health risks by heavy metal exposure.

Variable	Value
C_soil_ (mg kg^−1^): heavy metal concentration in soil	Table 2
ABS (unitless): dermal absorption factor	0.03 for As and 0.001 for other metals
AF (mg cm^−2^): soil to skin adherence factor	0.07 for Adults, 0.2 for Children
AT (d): averaging time	ED × 365 for non-carcinogenic and 70 × 365 for carcinogenic
BW (kg): body weight	70 for Adults, 15 for Children
ED (yr): exposure duration	30 for Adults, 6 for Children
EF (d yr^−1^): exposure frequency	350 for residential
ET (h d^−1^): exposure time	24 for residential
RfD (mg kg^−1^ d^−1^): chronic reference dose	Supplementary Table 1
RI (mg d^−1^): ingestion rate	100 for Adults, 200 for Children
PEF (m^3^ kg^−1^): soil-to-air particle emission factor	1.36 × 10^9^
SA (cm^2^ event^−1^): skin surface area available per event of heavy metal exposure	5700 for Adults, 2800 for Children
SF (mg kg^−1^ d^−1^): carcinogenicity slope factor	Supplementary Table 2

Chronic daily intake (*CDI*):

(2) $${CDI}_{Ing}=\frac{C_{soil}\times RI\times EF\times ED}{BW\times AT}{\times 10}^{-6}\text{,}$$

(3) $${CDI}_{Derm}=\frac{C_{soil}\times SA\times AF\times ABS\times EF\times ED}{BW\times AT}\times 10^{-6}\text{,}$$

(4) $${CDI}_{Inh}=\frac{C_{soil}\times EF\times ET\times ED}{PEF\times BW\times AT}$$

Non-carcinogenic risk:

(5) $$HQ=\frac{CDI}{RfD}$$

$$HI=\sum HQ={HQ}_{Ing}+{HQ}_{Derm}+{HQ}_{Inh}$$

(6) $$={\left(\frac{CDI}{RfD}\right)}_{Ing}+{\left(\frac{CDI}{RfD}\right)}_{Derm}+{\left(\frac{CDI}{RfD}\right)}_{Inh}$$

Carcinogenic Risk:

(7) CR=CDI×SF

$$LCR=\sum CR={CR}_{Ing}+{CR}_{Derm}+{CR}_{Inh}$$

(8) $$={\left(CDI\times SF\right)}_{Ing}+{\left(CDI\times SF\right)}_{Derm}+{\left(CDI\times SF\right)}_{Inh}$$

## 3 Results and discussion

### 3.1 Heavy metal concentration and source identification

The soil and sediment samples were collected and analyzed by Samaniego et al. (2021) [12] from the following areas in the study site: PQMI, Tagburos River, wharf, Honda Bay, surrounding soils and sediments, and other rivers. The descriptive statistics of the heavy metals in the soils and sediments are summarized in Table 2. The range of the mean heavy metal concentrations are As (4.2–50.7 mg kg^−1^), Ba (7.5–52.1 mg kg^−1^), Cd (0.2–0.3 mg kg^−1^), Co (27.7–124.7 mg kg^−1^), Cr (205–1480 mg kg^−1^), Cu (50.7–116.9 mg kg^−1^), Fe (2.96–16.2%), Hg (1.6–397.2 mg kg^−1^), Mn (268–2559 mg kg^−1^), Ni (362–2531 mg kg^−1^), Pb (10.5–36.4 mg kg^−1^), Sb (0.8–30.2 mg kg^−1^), Tl (0.1–20.9 mg kg^−1^), V (27.5–159.9 mg kg^−1^), and Zn (37–114 mg kg^−1^) [12]. PQMI and the wharf have the highest mean heavy metal concentrations, particularly Hg. In terms of individual heavy metal, PQMI has the highest concentrations of Co, Cr, and Ni while the wharf has the highest concentrations of As, Cd, Cu, Hg, Pb, Sb, Tl, and Zn. It is also notable that the mean As, Cd, Co, Cr, Cu, Hg, Ni, Sb, and Tl concentrations are equivalent to 3.1, 2.5, 5, 8.3, 2.6, 2113.7, 27, 17.6, and 4.3 times of the mean concentrations in the upper continental crust [32]. The Hg in the study area is certainly sourced from historical Hg mining by PQMI. As mine waste calcine has been seen as the largest source of Hg contamination in the abandoned mine due to the low efficiency of Hg recovery during calcination [10], what is intriguing is the existence of other toxic metals with it.

**Table 2:** Mean heavy metal concentrations found in the sampling areas (mg kg^−1^) analyzed by Samaniego et al. (2021).

Location	PQMI	Tagburos River	Wharf	Honda Bay	Surrounding soils and sediments	Other rivers
*n*	*4*	*9*	*3*	*6*	*6*	*4*
As	7.75	4.89	50.67	16.33	5.75	5.75
Ba	29.5	52.11	29.33	7.5	38.33	47.75
Cd	0.21	0.28	0.34	0.21	0.16	0.175
Co	124.7	98.52	27.7	33.67	111	119.25
Cr	1479.75	513.33	1164.33	205	731.67	514.75
Cu	58.72	83.75	116.87	60.28	50.72	67.45
Fe	138,700	76,700	161,500	29,600	108,100	91,725
Hg	124.5	36.7	357.3	3.5	7.4	10.5
Mn	860.5	2559.44	267.67	270.67	1751	1922.75
Ni	2530.75	904.22	414.67	361.67	1375.17	2019.75
Pb	18.5	33.67	36.4	10.52	11.95	10.88
Sb	3.38	2.46	30.18	4.32	0.77	1.15
Tl	1.88	0.15	20.88	0.08	0.14	0.17
V	103	159.89	112.67	27.5	135.5	140
Zn	99.25	109.22	114	37	63.33	88

To better understand the phenomenon, PCA was carried out to identify the sources of the heavy metals. PCA is a useful tool for finding the causes of heavy metal pollution by grouping them according to similarity in their sources [13,17,19,[33], [34], [35], [36]]. PCA reduced the dimensionality of the dataset into five principal components that control the heavy metal pollution in the study area. Three of the five principal components have eigenvalues greater than 1 that control 95.9% of the total variance in the dataset. The eigenvalues, proportion of variance, probable sources, and heavy metal contribution of the PCs are summarized in Table 3. Table 4 provides a summary of the source apportionment or the locations where the PCs are distributed. The PC with the biggest proportion of variance, PC1 is responsible for most of the Sb, Tl, As, and Hg and is therefore identified as the mine waste calcine from the abandoned mine. As the calcine was dumped in Honda Bay to construct the wharf, the wharf gets the biggest apportionment by PC1 as shown in Table 4. PC2 is responsible for most of the V, Ba, Zn, and Co that are mostly apportioned to the Honda Bay. Since there are no industries or other sources that can probably be the source of heavy metal pollution in Honda Bay, we suspect that PC2 is related to leaching of the mobile heavy metals from the calcine in the wharf. For instance, Co, V, and Zn are mobile heavy metals under oxidizing and acidic soil conditions [37]. This hypothesis is supported by Ref. [38] which indicated that the calcine in the wharf is the source of heavy metal contamination in 10.13039/100004764Honda Bay. Thus, PC2 can also attributed to the abandoned mine. PC3 mostly provides Cr, Ni, Mn, and Fe which are all related to the geology of the study area. The study area belongs to the mantle component of the Palawan Ophiolite Complex that is dominated by harzburgites with few Cr-rich spinel [39,40].

**Table 3:** Eigenvalues, proportion of variance, probable sources, and heavy metal contribution of the five principal components identified by the PCA.

	PC1	PC2	PC3	PC4	PC5
Eigenvalues	7.82	4.41	2.15	0.44	0.18
Proportion of Variance (%)	52.12	29.37	14.36	2.97	1.19
Source	Abandoned mine	Abandoned mine	Natural	Other source	Other source
As	11.48	1.50	0.02	7.27	1.82
Ba	0.35	18.33	6.24	5.99	1.88
Cd	10.34	0.11	5.26	16.62	0.04
Co	5.32	11.69	3.16	0.20	0.03
Cr	3.03	4.91	23.11	6.11	12.31
Cu	10.72	0.45	5.32	0.22	15.04
Fe	5.01	7.14	11.27	7.62	9.58
Hg	11.13	0.36	5.25	0.20	0.13
Mn	3.54	10.68	11.50	0.32	2.11
Ni	2.67	7.84	18.08	1.00	29.00
Pb	7.87	3.11	6.62	19.86	9.45
Sb	12.28	0.23	0.01	6.54	0.23
Tl	12.13	0.00	0.20	10.55	0.02
V	0.01	19.59	3.80	10.11	5.07
Zn	4.11	14.05	0.16	7.41	13.29

**Table 4:** Source apportionment analysis.

Location	PC1	PC2	PC3	PC4	PC5
PQMI	0.19	5.46	51.47	26.17	0.03
Tagburos River	0.27	13.32	46.57	21.58	1.60
Wharf	77.63	0.08	0.00	5.62	0.00
Honda Bay	2.78	75.38	0.91	3.52	0.75
Surrounding soils	10.06	0.45	0.92	24.37	47.54
Other rivers	9.07	5.31	0.12	18.74	50.09

Ultramafic rocks like the harzburgites have Cr and Ni concentrations reaching 2980 mg kg^−1^ and 10,900 mg kg^−1^, respectively [41,42]. Meanwhile, Mn is related to the occurrence of red chert and dark manganiferous chert located northeast of the study area [43]. Majority of PC3 or the natural source is apportioned to PQMI pit lake and Tagburos River. This can be explained by the occurrence of the ultramafic rocks upstream of the Tagburos River that significantly affects the chemistry of PQMI soils and Tagburos River causing enrichment of Cr, Ni, and Mn [10]. Lastly, PC4 and PC5 were ascribed to other sources as we could not identify primary sources of Pb and Cd in the study area as well as other sources for Ni other than the calcine and the geology. These PCs are mostly apportioned to PQMI, Tagburos River, surrounding soils, and other rivers as shown in Table 4.

### 3.2 Ecological and health risks associated with heavy metals

**Fig. 2:**
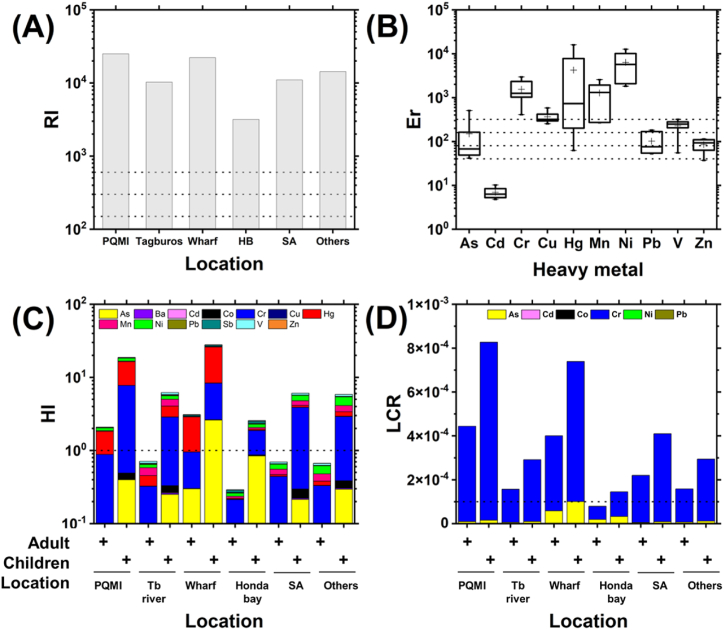
The ecological risk was assessed using the potential ecological risks index (RI). (A) RI shows that all the areas have highly strong ecological risks. (B) The statistics of ER of the individual heavy metals indicate that Ni, Hg, Cr, and Mn posed the highest ecological risk. Meanwhile, the human health risks were assessed using the hazard index (HI) and lifetime cancer risk (LCR). (C) HI suggests a strong potential for children in all the sampling sites to develop non-carcinogenic effects due to heavy metal exposure as well as for adults living near PQMI and the wharf. (D) The LCR for adults and children (except for adults on HB coast) exceeded the 10^−4^ limit suggesting a very strong potential for developing cancer. Some locations are abbreviated as follows: Tagburos river (Tb river), surrounding soils and sediments (SA), and other rivers (Others). Please refer to Fig. 1A for their locations. The dotted lines indicate the different pollution levels.

To assess the ecological and human health risks associated with the toxic metals, various indices were applied as these are currently the most widely used in many studies [31,[44], [45], [46], [47], [48]]. According to RI, all the sampling areas have highly strong ecological risks (Fig. 2A). The mean ER of the individual metals show that Ni (6339), Hg (4227), Cr (1536), Mn (1272), and Cu (365) presented a very high risk; V (226) has high risk; As (149), Pb (102), and Zn (85) have considerable risk; and Cd (7) has low risk (Fig. 2B). On average, the contribution of the heavy metals to the ecological risks is in the order of Ni (49.1%) > Hg (19.9%) > Mn (11.3%) > Cr (11.0%) > Cu (3.7%) > V (1.9%) > As (1.5%) > Pb (0.9%) > Zn (0.7%) > Cd (0.1%).

HI measures the potential non-carcinogenic effects of heavy metal exposure. The HI for adults ranged from 0.29 (Honda Bay) to 3.08 (wharf) while it ranged from 2.56 (Honda Bay) to 27.86 (wharf) for children. As a rule, the higher values of HI and HQ above 1 suggest a higher level of concern. The result of the modeling suggests the probability of adverse effects to adults and children in residents living nearby PQMI and the wharf as well as to children in Honda Bay, surrounding areas, around Tagburos River, and near other rivers (Fig. 2C). It was noted that children are nine times more vulnerable to the non-carcinogenic effects of heavy metals than adults. The mean HQs of Cr and Hg for children alone are much greater than one. The mean HQ contribution of the heavy metals to the overall HI for both adults and children is in the order of Cr (40.24%) > Hg (23.38%) > Ni (10.6%) > As (9.6%) > Mn (8.1%) > V (3.7%) > Sb (2.2%) > Pb (1.0%) > Co (0.8%) > Cu (0.3%) > Ba (0.1%) > Cd (0.06%) > Zn (0.05%).

The LCR for both adults and children in all the sampling areas (except for adults in Honda Bay) all exceeded 10^−4^, suggesting a potentially great risk for developing cancer (Fig. 2D). The LCR for adults ranged from 7.89 × 10^−5^ – 4.44 × 10^−4^ and for children 1.45 × 10^−4^ – 8.27 × 10^−4^. The mean CR contribution of the heavy metals to LCR for both adults and children is Cr (91.8%) > As (8.1%) > other heavy metals (0.1%). The results of the health risk analyses differ significantly from the work of [43] that discounted in their calculations the carcinogenic effects of Co, Cr, and Ni. Surprisingly, it was found that these three heavy metals contribute 92% to the LCR for both adults and children. Their previous work also used the 95% UCL concentrations of the heavy metals in the entire study area instead of basing them according to sampling vicinity which led to underestimation of potential public health risks. It should be noted that [43] reported a different number of sampling points despite the similar sampling dates reported in their previous paper [12] which could also have affected the outcome of the health risk modeling. The detailed contribution (%) of the individual heavy metals to the overall RI, HI, and LCR by specific location is presented in Table 5.

**Table 5:** Contribution (in %) of the individual heavy metals to the ecological risk index (RI), hazard index (HI), and lifetime cancer risk (LCR) at specific sampling areas. Since there is a negligible difference in the contribution by age group (<±0.2%), the values are presented as averages. Distinctly high values that contribute significantly to the risk indices are highlighted in bold. LCR from Ba, Cu, Hg, Mn, Sb, V and Zn were not calculated because these metals are not carcinogenic. Some locations are abbreviated as follows: Tagburos river (Tb river), surrounding soils and sediments (SA), and other rivers (Others). Please refer to Fig. 1A–C for their relative locations.

Heavy metal	Location	Potential Ecological Risk Index (RI)	Hazard Index (HI)	Lifetime Cancer Risk (LCR)
As	PQMI	0.31	2.16	1.92
	Tb river	0.48	4.06	3.44
	Wharf	2.28	9.52	**14.02**
	Honda Bay	5.16	**32.95**	**22.96**
	SA	0.38	3.54	2.09
	Others	0.40	5.09	4.01
Ba	PQMI		0.03	
	Tb river		0.17	
	Wharf		0.02	
	Honda Bay		0.06	
	SA		0.12	
	Others		0.16	
Cd	PQMI	0.03	0.02	0.00
	Tb river	0.08	0.07	0.00
	Wharf	0.05	0.02	0.00
	Honda Bay	0.20	0.14	0.00
	SA	0.04	0.04	0.00
	Others	0.04	0.05	0.00
Co	PQMI		0.43	0.02
	Tb river		1.02	0.05
	Wharf		0.06	0.01
	Honda Bay		0.84	0.03
	SA		1.17	0.04
	Others		1.31	0.06
Cr	PQMI	**11.84**	**39.07**	**98.00**
	Tb river	**10.03**	**40.40**	**96.36**
	Wharf	**10.50**	**20.73**	**85.92**
	Honda Bay	**12.95**	**39.22**	**76.91**
	SA	**13.32**	**58.86**	**97.80**
	Others	7.21	**43.18**	**95.81**
Cu	PQMI	1.17	0.10	
	Tb river	4.09	0.42	
	Wharf	2.63	0.13	
	Honda Bay	9.52	0.74	
	SA	2.31	0.26	
	Others	2.36	0.36	
Hg	PQMI	**31.00**	**45.35**	
	Tb river	**10.32**	**18.43**	
	Wharf	**71.63**	**62.71**	
	Honda Bay	1.97	2.65	
	SA	1.84	3.60	
	Others	2.84	7.54	
Mn	PQMI	3.44	1.89	
	Tb river	**25.00**	**16.72**	
	Wharf	1.21	0.40	
	Honda Bay	8.55	4.30	
	SA	**15.94**	**11.70**	
	Others	**13.47**	**13.39**	
Ni	PQMI	**50.62**	8.58	0.04
	Tb river	**44.17**	9.14	0.04
	Wharf	9.35	0.95	0.01
	Honda Bay	**57.10**	8.89	0.03
	SA	**62.59**	**14.21**	0.04
	Others	**70.72**	**21.76**	0.08
Pb	PQMI	0.37	0.36	0.02
	Tb river	1.64	1.96	0.11
	Wharf	0.82	0.48	0.05
	Honda Bay	1.66	1.49	0.07
	SA	0.54	0.71	0.03
	Others	0.38	0.68	0.04
Sb	PQMI		0.66	
	Tb river		1.44	
	Wharf		3.98	
	Honda Bay		6.13	
	SA		0.46	
	Others		0.72	
V	PQMI	0.82	1.32	
	Tb river	3.12	6.10	
	Wharf	1.02	0.97	
	Honda Bay	1.74	2.55	
	SA	2.47	5.28	
	Others	1.96	5.69	
Zn	PQMI	0.40	0.02	
	Tb river	1.07	0.07	
	Wharf	0.51	0.02	
	Honda Bay	1.17	0.06	
	SA	0.58	0.04	
	Others	0.62	0.06	

The samples analyzed in this study included both soils and sediments (from rivers). It makes sense to assess the risks that heavy metals in soil pose to the environment and to human health because of its accessibility. However, it is significant to note that the risks by heavy metals in the sediments needs to be properly considered. Other authors provided a caveat that compared to soil, river sediments are difficult for humans to access and thus risk calculation may be overestimated [49,50]. Nonetheless, sediment samples have been included in the study as it is culture in the Philippines for communities to do various activities in riverbanks such as bathing, laundry, shellfish picking, and the like.

Overall, the heavy metals such as Cr and Ni that are mainly coming from natural sources (PC3) contributed most to the mean ecological and human health risks of the study area. Intuitively, the risks due to heavy metal exposure can all be attributed to the mining site, neglecting the potential impact by other heavy metal sources. In the case of the abandoned PQMI mine in Palawan, such disregard would lead to inaccuracy as it was shown that, on average, the heavy metals from natural sources (i.e., local geology) contributed more to the risks than the Hg coming from the mine. Despite having 8 times less Tr that the Hg, Ni has higher mean concentrations [12], thus it contributed most to the RI. Moreover, Cr has higher RfD for ingestion and dermal pathways while at the same time has higher mean concentrations than the Hg.

### 3.3 Estimating the contribution of the heavy metal sources to the risks

The mine had not undergone rehabilitation even after its abandonment in 1976. More than a million tons of mine waste calcines produced by the mercury mine was dumped in Honda Bay to construct the wharf [10,51]. The dilemma of heavy metal contribution to health risks in the area is further compounded by the island's geology which is largely ultramafic rocks that contain high concentrations of Cr and Ni [39,40]. Knowledge on the role of these heavy metal pollution sources to the ecological and human health risks remains unknown.

Given the challenge in understanding how the complex origins of the heavy metals contribute to specific health and ecological risks, we integrated the PCA results with the contribution of individual heavy metals to the risks. The following should be noted: (a) PC1 and PC2 are both associated to the contribution of the abandoned mine since the former contributes the most Hg and the latter has the possibility of leaching of mobile metals due to erosion; (b) PC3 is attributed to natural sources due to high apportionment of Cr, Ni, Mn, and Fe; and (c) the remaining PC4 and PC5 can be ascribed to other “unknown” sources. From this interpretation, we then calculated the contribution of the individual heavy metals from particular PCs to the risk using Equation (9):

(9) $$Contribution\;\left(\%\right)=Elemental\;contribution\times Source\;apportionment\times {Risk\;contribution\times 10}^{-3}$$

The elemental contribution in Table 3 was multiplied to the source apportionment in Table 4 and the risk contribution in Table 5 and multiplied by 10^−3^. The final estimated contribution by a PC was calculated by normalizing to the sum of the heavy metal contributions of the PCs calculated using Equation (9). A sample computation for the contribution of the PCs to HI in the wharf is presented in Table 6.

**Table 6:** The contribution of the PCs to HI in the wharf was calculated by multiplying the heavy metal contribution of the PCs in Table 2, the source apportionment in Table 3, and the heavy metal contribution (%) to the HI provided in Table 4, divided by 10^3^. Note that PCs 3 and 5 have negligible contributions.

Heavy metals	PC1	PC2	PC4	
As	8.48		0.39	
Ba				
Cd	0.02			
Co	0.03			
Cr	4.88	0.01	0.71	
Cu	0.11			
Hg	54.19		0.07	
Mn	0.11			
Ni	0.20		0.01	
Pb	0.29		0.05	
Sb	3.80		0.15	
V			0.06	
Zn	0.01			
Total	72.11	0.01	1.44	73.55
Final Contribution	98.03	0.02	1.95	100

**Fig. 3:**
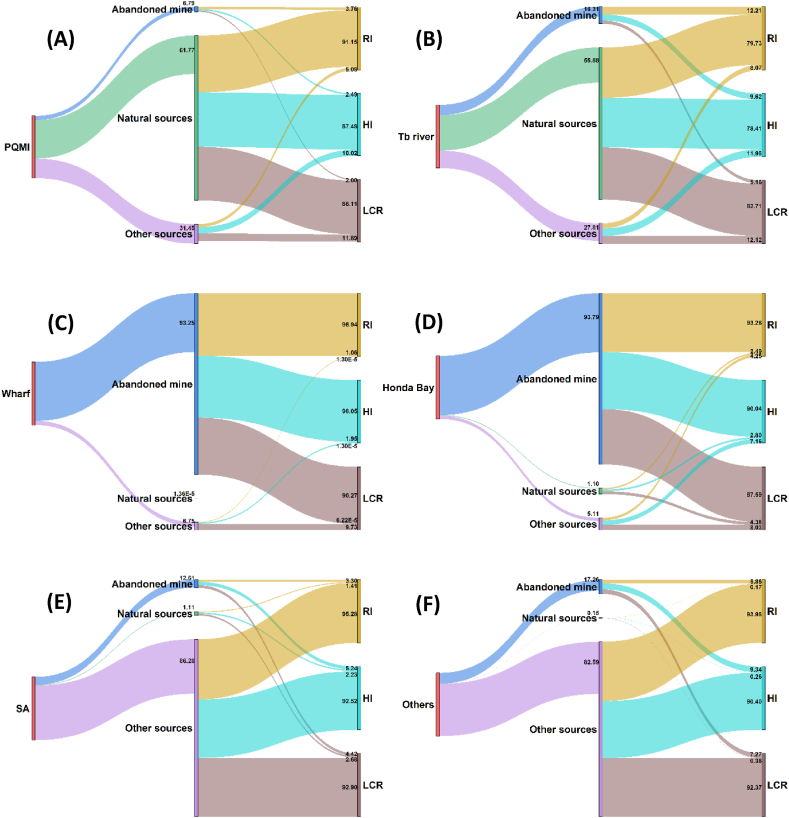
Sankey diagrams showing the estimated contributions of heavy metals from the abandoned mine, natural sources (geology), and other unidentified sources to the overall RI, HI, and LCR. Shown are the scenarios of apportionment and risk contribution in (A) PQMI, (B) Tagburos river (Tb river), (C) Wharf, (D) Honda bay, (E) surrounding soils and sediments (SA), and (F) other rivers (Others). Please refer to Fig. 1A for their locations.

By doing the computation for all the sampling locations, the contribution of the abandoned mine, natural source, and other sources to RI, HI, and LCR are presented in Fig. 3. It is necessary to evaluate the method's effectiveness. Even though it may be difficult or impossible to pinpoint exactly how each pollution source contributes to the risks, we give compelling explanations based on the findings of the risk assessments and prior knowledge of the study area.

Using Sankey diagrams, Fig. 3A to F illustrate how the contribution of the three sources (abandoned mine, natural and unidentified) weighed in on the health and ecological risks at each study site. Based on our analysis, the abandoned mine contributed most to all the risks in the wharf and Honda Bay (Fig. 3C and D). This makes sense as Hg and Cr from the abandoned mine contributed most to the risks in the wharf. Of note, the calcines were dumped in Honda Bay to construct the wharf and it is probable that some of the mobile heavy metals were being leached from the calcine to the bay. In Honda Bay, we showed that As, Cr, and Ni contributed the maximum risks. Notably, calcine also contain huge concentrations of Cr and Ni [12]. In contrast, the role of natural sources was most prominent at PQMI and Tagburos River as shown in Fig. 3A and B. The most influential metals at PQMI and Tagburos River were Cr and Ni which are apparently from geogenic materials. Most of the heavy metals coming from the natural sources are apportioned to these locations (see Table 3, Table 4). Interestingly, these results are consistent with source apportionment shown in Table 4.

### 3.4 Policy recommendations for rehabilitation and mitigation of environmental impact

The abandonment of the PQMI Hg mine in 1976 was a lost opportunity for proper mine rehabilitation causing the mine wastes to be exposed to the environment [51] and vice-versa. Despite the danger of Hg mining and processing, strong economic activity associated with it resulted to rapid urbanization in the area. The area surrounding the abandoned mine is home to more than 12,200 people as of 2015. Our findings suggest a very strong potential to cause adverse health effects to residents due to heavy metal exposure which is supported by recent work [43]. Here, we present some key recommendations that the government through the Department of Environment and Natural Resources (DENR) and the local government of Palawan can adopt to mitigate the harmful effects of the heavy metals on the environment and the population:

• The land use plan in the affected areas should be reviewed to mitigate potential harm to the community without affecting key sources of employment. The local government should commit to achieving environmental justice through fair treatment and meaningful involvement of all people in decisions that affect public health and the environment. It is also reasonable for the residents who developed illnesses induced by toxic metals to be compensated.
• Planting food crops that accumulate heavy metals should be avoided. The absorbed toxic heavy metals may end up in the food chain causing toxic effects on humans. In previous studies, vegetable consumption accounts for 90% of heavy metal exposure in humans [[52], [53], [54]]. The capacity of plants to accumulate heavy metals also differs by heavy metal [[55], [56], [57], [58]]. In general, leafy vegetables have higher heavy metal uptake than non-leafy vegetables [51,59]. Future studies on bioaccumulation of heavy metals in food crops as well as fish products from Honda Bay can be pursued.
• As Palawan is a growing hub for shrimp aquaculture, deforestation of mangroves should be minimized since shrimps grow in these areas [[60], [61], [62], [63]]. Since mangroves located at the mouth of the Tagburos River act as the heavy metal filter at Honda Bay [64], the mangrove density must be enhanced.
• Intervention engineering of the wharf to prevent the heavy metals from seeping into the environment. Activities that can cause the mobilization of heavy metals in the wharf such as major construction and dredging must also be prohibited.
• Market-based incentives necessary for reducing and rehabilitation of heavy metal-polluted areas can be devised. Market incentives not limited to pollution fees and pollution taxes can be developed and enforced in industries in Palawan. As tourism draws the immense income from the region, a larger portion of the revenue can be allocated for Honda Bay rehabilitation.

## 4 Conclusions

The *admixtures* of heavy metals in soils and sediments surrounding the PQMI abandoned Hg mine in Palawan, Philippines are several times higher than the mean concentrations in the upper continental crust. The results of the multivariate analysis (PCA) demonstrate that the sources of heavy metals can be traced to the mine waste calcine, the local geology (i.e., ultramafic rocks), and other unidentified sources. The inconsiderate use of the calcine as land filler and in the construction of the wharf led to the exposure of heavy metals. These heavy metals pose a highly strong ecological risk mainly by Ni, Hg, and Cr. Further, both the elevated non-carcinogenic and carcinogenic health risks are caused mainly by Cr which is sourced from all the sources. The other risk contributors such as Hg and As from the abandoned mine are just secondary. The contribution of the pollution sources by combining the PCA results and the risk assessments was carried out in this work. It was estimated that the abandoned mine contributed to bulk of the risks in the wharf and Honda Bay. While the natural sources appear to be solely responsible for heavy metal contamination in PQMI pit lake and Tagburos River, and the other unidentified sources in surrounding soils and other rivers, the impact of the abandoned mine in these sampling areas cannot be neglected as described by Samaniego et al. (2020, 2021) [10,12]. Additionally, the possibility that the calcine from the abandoned mine is the unidentified source cannot be ruled out, as it was previously stated that the calcine was used as a land filler in nearby settlements.

Since the analysis was limited to total Cr, it is recommendable to study the speciation of the Cr surrounding the abandoned mine as Cr (VI) poses greater environmental and health risks. Even though this study employed the slope factor of 0.5 for Cr (VI), it was done so to avoid underestimating the carcinogenic risk in a manner that would be consistent with previous research [31,46,47,[65], [66], [67], [68], [69], [70]]. It is also recommended to study the properties of the soils and sediments (i.e., total organic content and clay content) since these can affect the enrichment of the heavy metals [71]. Moreover, only data that were clustered sampling points were available for the PCA.

Lastly, this study has profound implications in environmental policy development not only for the local government of Palawan but also for other mined-out sites. While the study by Samaniego et al. (2021) [12] analyzed heavy metals occurring with the Hg, this study addressed other relevant topics such as identification of the sources of these metals and estimation of their contributions to ecological and human health risks. This study also upholds the importance of non-biased approach in the estimation of the risks. Given that the Philippines has recently ratified the Minamata Convention on Mercury, this study supports a conservative framework for risk assessment by heavy metal exposure to help protect the general population. It is recommended that the local government of Palawan implement an information campaign about the contribution of these heavy metal sources to the outcome of the ecological and health risks. There is a need to manage any risk of miscommunication that may cause confusion and panic to the residents as well as tourists.

## Author contribution statement

Lawrence Phoa Belo; Custer C Deocaris; Lawrence Phoa Belo: Conceived and designed study; Analyzed and interpreted the data; Contributed, materials, analysis tools or data.

Reymar R. Diwa, Custer C Deocaris, Lawrence Phoa Belo; Lhevy Geraldo: Wrote the paper.

Reymar R. Diwa, Lhevy Geraldo: Performed calculations .

## Data availability

The datasets generated during and/or analyzed during the current study are available in this published article by Samaniego et al. (2021) https://doi.org/10.29037/ajstd.682. All other data resulting from the analysis will be made available upon request.

## Declaration of competing interest

The authors declare that they have no known competing financial interests or personal relationships that could have appeared to influence the work reported in this paper.
