# Supplementary material for: Ecological and health risks from heavy metal sources surrounding an abandoned mercury mine in the island paradise of Palawan, Philippines
Source: Heliyon. 2023 Apr 28;9(5):e15713. doi: 10.1016/j.heliyon.2023.e15713 (PMC10256827; doi:10.1016/j.heliyon.2023.e15713)
Supplement: Multimedia component 1 [file mmc1.docx]

**Ecological and health risks from heavy metal sources surrounding an abandoned mercury mine in the island paradise of Palawan, Philippines**

Reymar R. Diwa ^1,2^, Custer C. Deocaris ^2,5^, Lhevy D. Geraldo ^3^ and Lawrence P. Belo ^4,5,^*

*^1^ Research and Development Center, Rizal Technological University, Mandaluyong City 1550, Philippines;* [*rdiwa@rtu.edu.ph*](mailto:rdiwa@rtu.edu.phcom) *(R.D.)*

*^2^ Atomic Research Division, Philippine Nuclear Research Institute, Department of Science & Technology, Diliman, Quezon City 1101, Philippines;* [*ccdeocaris@pnri.dost.gov.ph*](mailto:ccdeocaris@pnri.dost.gov.ph) *(C.D.)*

*^3^ Earth and Space Sciences Department, College of Arts and Sciences, Rizal Technological University, Mandaluyong City 1550, Philippines;* [*ldgeraldo@rtu.edu.ph*](mailto:ldgeraldo@rtu.edu.ph) *(L.G.)*

*^4^ Department of Chemical Engineering, De La Salle University, Manila 1004, Philippines;*

*^5^ BANToxics, Barangay Central, Quezon City, 1100 Philippines*

***** Correspondence: [lawrence.belo@dlsu.edu.ph](mailto:lawrence.belo@dlsu.edu.ph) (L.B.); Tel.: (+63917 8362356)

**APPENDIX**

**Supplementary Table** **1**. Chronic reference dose (RfD, mg kg^-1^ d^-1^) for different heavy metal exposure pathways for the assessment of non-carcinogenic risks (Diami et al., 2016; Doležalová Weissmannová et al., 2019; Luo et al., 2012; Samaniego et al., 2022).

| **Heavy metal** | **RfD Ing** | **RfD Derm** | **RfD Inh** |
| --- | --- | --- | --- |
| **As** | 3.00$\times$10^-4^ | 1.23$\times$10^-4^ | 3.01$\times$10^-4^ |
| **Ba** | 7.00$\times$10^-2^ | 4.90$\times$10^-3^ | 1.43$\times$10^-4^ |
| **Cd** | 1.00$\times$10^-3^ | 1.00$\times$10^-5^ | 5.70$\times$10^-5^ |
| **Co** | 2.00$\times$10^-2^ | 4.90$\times$10^-3^ | 1.43$\times$10^-4^ |
| **Cr** | 3.00$\times$10^-3^ | 6.00$\times$10^-5^ | 2.86$\times$10^-5^ |
| **Cu** | 4.00$\times$10^-2^ | 1.20$\times$10^-2^ | 4.00$\times$10^-2^ |
| **Hg** | 3.00$\times$10^-4^ | 2.10$\times$10^-5^ | 8.57$\times$10^-5^ |
| **Mn** | 4.60$\times$10^-2^ | 1.84$\times$10^-3^ | 1.43$\times$10^-5^ |
| **Ni** | 2.00$\times$10^-2^ | 5.40$\times$10^-3^ | 2.06$\times$10^-2^ |
| **Pb** | 3.50$\times$10^-3^ | 5.25$\times$10^-4^ | 3.45$\times$10^-3^ |
| **Sb** | 4.00$\times$10^-4^ | 8.00$\times$10^-6^ | - |
| **V** | 7.00$\times$10^-3^ | 7.00$\times$10^-5^ | 7.00$\times$10^-3^ |
| **Zn** | 3.00$\times$10^-1^ | 6.00$\times$10^-2^ | 3.00$\times$10^-1^ |

**Supplementary Table** **2**. Carcinogenicity slope factor (SF, mg kg^-1^ d^-1^) of the carcinogenic metals for different exposure pathways for the assessment of carcinogenic risks (Faiz et al., 2012; Kamunda et al., 2016; Samaniego et al., 2022).

| **Heavy metal** | **SF Ing** | **SF Derm** | **SF Inh** |
| --- | --- | --- | --- |
| **As** | 1.50$\times$10^0^ | 3.66$\times$10^0^ | 1.51$\times$10^1^ |
| **Cd** |  |  | 6.30$\times$10^0^ |
| **Co** |  |  | 9.80$\times$10^0^ |
| **Cr** | 5.00$\times$10^-1^ |  | 4.20$\times$10^-1^ |
| **Ni** |  |  | 8.40$\times$10^-1^ |
| **Pb** | 8.50$\times$10^-3^ | 4.30$\times$10^-2^ |  |
